# Bacterial biosynthetic superclusters: emerging drivers of synergistic natural products against antimicrobial resistance

**DOI:** 10.1042/EBC20250019

**Published:** 2026-08-19

**Authors:** Karla Alexa Pentón, Yvonne Mast, Beatriz Cámara

**Affiliations:** 1 Programa de Doctorado en Biotecnología, Pontificia Universidad Católica de Valparaíso and Universidad Técnica Federico Santa María, Valparaíso, Chile; 2 ActinoLab USM, Center of Biotechnology “Daniel Alkalay Lowitt” & Department of Chemistry, Universidad Técnica Federico Santa María, Avenida España 1680, 2390136 Valparaíso, Chile; 3 Leibniz Institute DSMZ - German Collection of Microorganisms and Cell Cultures GmbH, Inhoffenstraße 7B, 38124 Braunschweig, Germany

**Keywords:** Bacterial biosynthetic superclusters, Co-producing metabolites, Synergistic natural products

## Abstract

Antimicrobial resistance (AMR) is a critical threat to global health and the economy. A promising strategy to address this challenge is the exploration of synergistic combinations of natural antimicrobials, which can broaden antibiotic spectra, enhance efficacy, and reduce the likelihood of resistance development. Such combinations often arise in bacteria through complex metabolic pathways encoded by biosynthetic superclusters (BSs)—genomic arrangements in which multiple biosynthetic gene clusters (BGCs) co-localize within the same genomic region, promoting co-regulation and co-production of specialized metabolites with complementary activities. This review provides an overview of current knowledge on biosynthetic gene superclusters in bacteria, focusing on their genomic architectures, regulatory connectivity, and the synergistic interaction profiles of the encoded metabolites as shaped by evolutionary pressures. Finally, we highlight how integrative genome–metabolome mining can be applied to discover, validate, and reprogram supercluster genes, positioning them as promising, naturally evolved platforms for the rational design of modular antimicrobial combinations to help combat AMR.

## Introduction

Antimicrobial resistance (AMR) has emerged as a critical global health threat, diminishing the effectiveness of existing antibiotics and dramatically increasing morbidity, mortality, and economic burden worldwide, with nearly 5 million AMR-associated deaths reported in 2019 and projections reaching 10 million annually by 2050 [1]. This escalating crisis highlights the need for antibiotic discovery pipelines that integrate resistance management from the outset, positioning synergistic combination therapies as a promising approach to improve efficacy, broaden antimicrobial spectra, delay resistance development, and even restore the activity of antibiotics compromised by existing resistance [2]. Such strategies align with current regulatory priorities established by the US Food and Drug Administration and the European Medicines Agency, which emphasize the importance of rationally designed drug combinations [3,4]. However, only a handful of natural-product-based combinations have reached clinical use—most notably β-lactam/β-lactamase inhibitors [5] and streptogramin combinations [6]—underscoring a largely unexplored research space with considerable potential.

Remarkably, many clinically important antimicrobials originate from bacteria—particularly Actinomycetota—which continue to provide chemically diverse scaffolds for new therapeutic development [7]. These metabolites are encoded within discrete genomic regions known as biosynthetic gene clusters (BGCs), defined as physically clustered sets of genes that collectively direct the biosynthesis, modification, regulation, and export of natural products (NPs), and their chemical variants [8]. Structurally and biosynthetically, most bacterial NPs—particularly antimicrobial ones—fall into peptide- and polyketide-derived families, and are mainly synthesized by polyketide synthases (PKSs), non-ribosomal peptide synthetases (NRPSs), and ribosomally synthesized and post-translationally modified peptide (RiPP) pathways [9]. Advances in genome sequencing and genome mining have revealed not only an extraordinary diversity of BGCs [10], but also broadened our understanding of bacterial secondary metabolism beyond a BGC-centric view, revealing that biosynthetic elements can interact across numerous genomic levels—from individual genes to subclusters and entire BGCs—reflecting the complex organizational and regulatory architecture underlying NPs biosynthesis [11]. Indeed, several synergistic antimicrobial combinations have increasingly been associated with higher-order genomic arrangements, often referred to as a biosynthetic supercluster (BS), in which multiple BGCs coordinate the production of chemically distinct compounds that exhibit complementary bioactivities [12,13]. Although BSs have been relatively understudied, they are emerging as an advancing area of research.

## From single BGCs to BSs: a shift in the genomic paradigm

While the hallmark features of single BGCs are well established [8], BS represent a more complex form of multi-BGC genomic organization, whose structural and functional features have been clearly delineated in only a limited number of canonical cases [14–16]. Although sometimes described simply as co-localized or closely arranged BGCs, numerous examples demonstrate a deeper level of physical and functional interplay that cannot be explained by adjacency alone. Based on the bacterial BSs described to date, these arrangements are generally characterized by several recurrent features, namely (i) physical colocalization of two or more complete BGCs within a compact genomic region; (ii) coordinated coproduction of the metabolites encoded by these pathways, often spanning different chemical classes; (iii) evidence of coordinated regulation, frequently inferred from shared regulatory elements or synchronized transcriptional responses; and (iv) functional complementarity—in particular, synergistic interactions—between the resulting metabolites [15–29]. This framework provides a practical basis for analyzing these increasingly recognized genomic architectures and for understanding their implications for NP biosynthesis. According to published research, only a fraction of these antimicrobial co-localized multi-BGC regions in bacteria have been experimentally characterized to date, as summarized in Table 1. In line with this, recent genome-wide mining has identified various additional co-localized BGC pairs, encompassing antibiotic–antibiotic combinations as well as antibiotics co-localized with toxins, siderophores, metallophores, pigments, and others [30]. However, for most of these *loci* predicted to encode functionally complementary NP combinations, direct evidence for coordinated biosynthesis, integrated regulation, or bona fide synergistic activity remains largely unknown or understudied. This highlights the significant gap to understanding the functional integration within multi-BGC *loci*, which has yet to be experimentally resolved.

**Table 1:** Documented BSs in bacteria and their genomic and functional attributes

Compounds	Type of BGCs	Genomic architecture	Producing organism(s)	Synergistic Mechanism	Ref.
**(1)** Pristinamycin I and **(2)** Pristinamycin II	**(1)** NRPS, **(2)** PKS–NRPS	Interdigitated BGCs; interrupted by a cryptic PKS BGC	*Streptomyces pristinaespiralis*	Cooperative single-target binding	[15]
**(1)** Lankamycin and **(2)** Lankacidin	**(1)** PKS, **(2)** PKS–NRPS	Two BGCs in direct adjacency	*Streptomyces rochei*	Cooperative single-target binding	[16]
**(1)** Griseoviridin and **(2)** Viridogrisein	**(1)** PKS–NRPS, **(2)** NRPS	Two BGCs in direct adjacency	*Streptomyces spp.* (reported for *S. griseoviridis* and *S. niveoruber*)	Cooperative single-target binding	[17]
**(1)** Rapamycin and **(2)** Actinoplanic acids	**(1)** PKS–NRPS, **(2)** PKS–NRPS	Two BGCs in direct adjacency	*Streptomyces rapamycinicus, Streptomyces iranensis* and *Actinoplanes* sp. MA7066	Dual-target inhibition	[18]
**(1)** Sulfazecin and **(2)** Bulgecin	**(1)** NRPS, **(2)** Saccharide	Two BGCs in direct adjacency	*Paraburkholderia acidophila* and *Burkholderia ubonensis* subsp. *mesacidophila*	Dual-target inhibition	[19]
**(1)** Bacillomycin D and **(2)** Fengycin	**(1)** PKS–NRPS, **(2)** NRPS	Two BGCs in direct adjacency	*Bacillus subtilis* species complex (reported for *B. amyloliquefaciens* and *B. velezensis*)	Dual-target inhibition	[20]
**(1)** Laxaphycin A and **(2)** Laxaphycin B	**(1)** PKS–NRPS, **(2)** PKS–NRPS	Two BGCs in direct adjacency	*Scytonema hofmannii*	Multi-step inhibition	[21]
**(1)** Acidomycin, **(2)** Stravidins, **(3)** Dapamycins, and **(4)** α-methyl-KAPA	**(1)** PKS, **(2)** PKS–NRPS, **(3)** NRPS, **(4)** aa-derived precursor BGC	Four BGCs in direct adjacency	*Streptomyces* spp. (reported for *Streptomyces* sp. WAC05950)	Multi-step inhibition	[22]
**(1)** Cephamycin C and **(2)** Clavulanic acid	**(1)** NRPS, **(2)** Beta-lactam	Two BGCs in direct adjacency	*Streptomyces spp.* (reported for *S. clavuligerus, S. jumonjinensis*, and *S. katsurahamanus*)	Suppression of resistance	[23]
**(1)** Jessenipeptin and **(2)** Mupirocin	**(1)** NRPS, **(2)** PKS	Two BGCs in direct adjacency	*Pseudomonas* sp. QS1027	Enhanced penetration or delivery	[24]
**(1)** Anabaenolysin and **(2)** Cyclodextrins	**(1)** PKS–NRPS, **(2)** Saccharide	Two BGCs in direct adjacency	*Anabaena* spp. (reported for *Anabaena* sp. XSPORK2A, *Anabaena* sp. XPORK13A and *Anabaena* sp. XPORK15F)	Enhanced penetration or delivery	[25]
**(1)** Petrichorin A and **(2)** Petrichorin B	**(1)** NRPS, **(2)** NRPS	Two BGCs in direct adjacency	*Lentzea flaviverrucosa*	Not determined	[26]
**(1)** Borregomycin and **(2)** Anthrabenzoxocinone	**(1)** PKS, **(2)** PKS	“sandwich” architecture	*Streptomyces* sp. MST-144321	Not determined	[27]
**(1)** Arylomycin, **(2)** Globomycin, **(3)** Siamycin	**(1)** NRPS, **(2)** PKS–NRPS, **(3)** RiPP	Three BGCs in direct adjacency	*Streptomyces* sp. VB1	Not determined	[28]
**(1)** Clostrisin and **(2)** Cellulosin	**(1)** RiPP, **(2)** RiPP	Two BGCs in direct adjacency	*Clostridium cellulovorans*	Not determined	[29]

**Figure 1:**
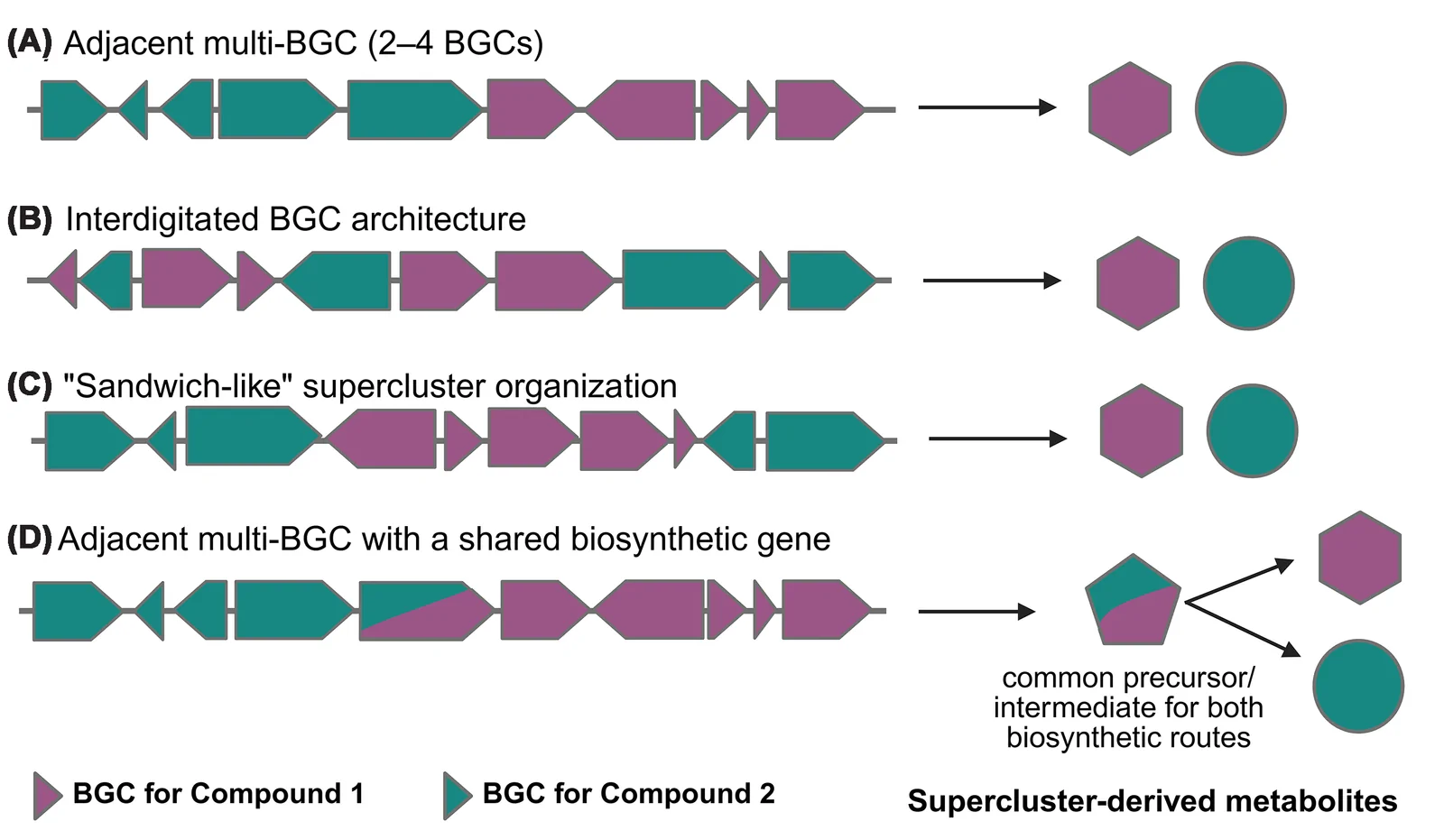
Genomic architectures of BSs and their co-produced metabolites Schematic representation of major genomic architectures observed in BSs and co-produced metabolites. (**A**) Directly adjacent architectures comprising 2–4 discrete and contiguous BGCs, as exemplified by the cephamycin C/clavulanic acid supercluster. (**B**) Interdigitated architecture, characterized by the interspersion of biosynthetic genes from distinct BGCs, producing a highly intermixed multi-BGC region, as exemplified by the pristinamycin supercluster. (**C**) Sandwich-like architecture, in which one BGC is positioned between two segments of another BGC, forming a nested genomic arrangement, as exemplified by the anthrabenzoxocinone–borregomycin supercluster. (**D**) Adjacent BGCs containing a shared biosynthetic gene whose encoded enzyme functions in both pathways, supplying a common precursor or intermediate and thereby underscoring metabolic integration within the supercluster, as exemplified by the laxaphycin A/B supercluster. Color-coded arrows denote individual BGCs, while color-matched symbols represent the metabolites co-produced by each pathway.

In general, BS exhibit diverse genomic architectures, varying widely in size, composition, and organizational complexity, as schematically summarized in the overview shown in Figure 1. Reported examples in bacteria range from relatively compact *loci* of ∼50 kb [31] to extensive regions exceeding 200 kb, with the pristinamycin supercluster [15] representing one of the largest and structurally most distinctive examples characterized so far. The most prevalent configuration consists of two autonomous BGCs arranged in direct adjacency (Figure 1A)—often separated by only a few kilobases [16–20,23,24,29]—although more complex assemblies containing three or even four BGCs have also been reported recently [22,28]. Beyond this canonical organization, more complex BSs have been described, in which genes encoding distinct biosynthetic pathways are distributed across the region rather than confined to clearly delimited, contiguous clusters (Figure 1B,C). This organization gives rise to partially overlapping or interdigitated assemblies, as illustrated by the pristinamycin (Figure 1B) [15] and anthrabenzoxocinone–borregomycin (Figure 1C) superclusters [27]. In the latter, the anthrabenzoxocinone PKS genes flank a centrally embedded borregomycin NRPS–PKS pathway in a distinctive “sandwich-like” arrangement [27]. A notable feature across most BS cases is that co-localized BGCs frequently belong to different biosynthetic classes—including PKS, NRPS, RiPPs, and hybrid pathways—suggesting that clustering is driven not by shared enzymatic lineage but rather by functional complementarity among the resulting metabolites [13]. Importantly, such physical proximity between multiple BGCs can itself promote chemical diversification through functional integration between pathways, ranging from metabolic cross-talk—as seen in the promiscuous cytochrome P450-mediated cross-linking during petrichorins A/B biosynthesis [26]—to enzyme-sharing mechanisms exemplified by the PKS-primed dual NRPS system of the laxaphycin supercluster [21] (Figure 1D).

To date, bacterial BSs have been identified in a relatively narrow range of genera, with a strong predominance in Actinomycetota—particularly *Streptomyces*—as summarized in Table 1. These examples encompass soil-derived producers such as *S. pristinaespiralis*, *S. rochei, S. griseoviridis*, *S. rapamycinicus*, and *Streptomyces* sp. MST-144321 [15–18,27], as well as the marine isolate *Streptomyces* sp. VB1 [28]. Additionally, a BS has been reported for *Lentzea flaviverrucosa*, a member of the so-called “rare actinomycete,” which are increasingly recognized as sources of chemically atypical metabolites [26]. Beyond Actinomycetota, further examples—including Cyanobacteriota, Pseudomonadota, and Bacillota [19–21,24,25,29]—show that superclusters can also arise in phylogenetically distant bacteria, inhabiting ecological niches shaped by strong selective pressures such as predation, interspecies competition, or resource limitation. Among the BSs discussed here, some are conserved across multiple bacterial species or genera, whereas others appear to have a more restricted taxonomic distribution. Well-supported examples include the β-lactam supercluster shared by all currently known cephamycin C/clavulanic acid-producing *Streptomyces* spp. [23]; the recurrent co-occurrence of the bacillomycin D and fengycin clusters within the *B. subtilis* species complex [32]; the colocalization of the actinoplanic acid cluster with the rapamycin BGC in rapamycin-producing actinobacteria [18]; the association of the anabaenolysin cluster with a candidate cyclodextrin-associated gene (*camA*) in anabaenolysin-producing *Anabaena* strains [25]; and the sulfazecin/bulgecin locus pair in two β-proteobacterial species [19]. In the streptogramin examples—including pristinamycin and griseoviridin/viridogrisein—conservation appears to be primarily functional, as distinct, yet analogous, genomic arrangements in *Streptomyces* producers nonetheless support the coordinated biosynthesis of complementary A/B components [15,17]. By contrast, most of the remaining BS appear, based on current evidence, to be more narrowly distributed. For example, the supercluster described in *Streptomyces* sp. VB1 has thus far been identified as a highly similar tripartite arrangement in only one closely related strain, while more distantly related *Streptomyces* genomes retain only partial combinations of the constituent subclusters [28]. Within these groups, Actinomycetota strains from terrestrial habitats frequently harbor especially large and gene-rich BGCs, a feature that may facilitate the emergence of BS arrangements. Nonetheless, the major BGC classes occur across all ecosystems and phyla [9], indicating that the genomic capacity for complex biosynthesis is not restricted to particular lineages. Altogether, the current taxonomic bias in reported superclusters likely reflects the historical emphasis on soil-derived *Streptomyces* in genome sequencing, metabolomic profiling, and antibiotic research rather than a true lineage-specific constraint. As genome mining efforts increasingly expand toward underexplored taxa and more high-quality assemblies and metabolome-resolved workflows become available, the apparent taxonomic range of BS is likely to broaden substantially. In general, conceptual analyses suggest that such multi-BGC arrangements may represent an overlooked but evolutionarily widespread strategy by which bacteria encode synergistic chemistry at the genomic level, positioning co-localized BGCs as a privileged search space for bioactive combinations that conventional screening approaches often overlooks [12].

Coordinating the activity of multiple BGCs is a defining and intrinsically complex feature of BS, relying on hierarchical regulatory networks that span pathway-specific to global levels of control [33]. Characterized bacterial BS often encode a diverse and large repertoire of regulatory genes, many of which function as cluster-situated regulators (CSRs)—pathway-specific transcription factors encoded within individual BGCs or embedded in the interstitial regions of the BS—where they exert direct and localized transcriptional control over one or multiple biosynthetic modules [34–36]. CSRs largely belong to well-established regulatory families, including SARP activators [34,37,38], LuxR-type regulators, TetR-like repressors [17,39], and two-component systems [40,41], which collectively provide fine-tuned regulation of individual BGCs pathways or coordinated segments of the BS. Beyond this local layer, BS expression can also be modulated by global or pleiotropic regulators, such as anti–anti-sigma factor BldG—that influences the β-lactam supercluster by controlling the transcription of the SARP activator gene *ccaR* in *S. clavuligerus* [42]—as well as by quorum-sensing-like γ-butyrolactone signaling-molecule/receptor systems [43,44]. In contrast, in *Bacillus* species, fengycin-bacillomycin D supercluster—and the closely related surfactin pathway—are directly and coordinately regulated through shared global regulatory networks, involving CodY, DegU, ComA, and Spo0A, integrating quorum-sensing stress, and, developmental signals in soil- and plant-associated environments [45]. Nevertheless, to date only a handful of BSs have been comprehensively characterized in terms of their regulatory mechanisms, most notably the β-lactam [35] and streptogramin [17,34] superclusters—underscoring how limited our current knowledge is of transcriptional coordination across multi-BGC *loci*.

## Functional logic: evolving to natural synergy within superclusters

Beyond their genomic architecture, the functional significance of BSs lies in the diverse activity interaction profiles exhibited by their co-produced metabolites. These interactions range from synergistic, where the combined effect of the co-produced compounds exceeds the additive sum of their individual effects, to antagonistic interactions, where one metabolite diminishes or counteracts the activity of another [46]. Instead, contingent interactions occur when two or more metabolites fulfill overlapping ecological functions under distinct environmental conditions, providing context-dependent redundancy that can enhance niche adaptation [11,47]. Current evidence suggests that bacterial superclusters often encode metabolite ensembles with synergistic activities [11], which may contribute to diverse ecological functions related to cross-kingdom interactions, microbial competition, and niche adaptation. Notably, the amoeba–associated *Pseudomonas* sp. QS1027 strain harbors a mupirocin-jessenipeptin supercluster that encodes synergistic amoebicidal metabolites, thereby contributing to resistance against predation by the bacterivorous amoeba *Dictyostelium discoideum* [24]. In a comparable context, the fengycin-bacillomycin D supercluster in *Bacillus* spp. has been linked to beneficial plant-associated interactions through synergistic antifungal activity against *Fusarium oxysporum* [20], supporting the idea that antagonistic ecological interactions may promote the evolution of BS as a strategy to enhance competitive fitness [47,48]. In addition, some supercluster-associated metabolite combinations have been proposed to confer selective advantages, such as the cephamycin–clavulanic acid pair against β-lactamase-producing bacteria [49] and the recently described ubiquitous anti-biotin supercluster, which has been suggested as a conserved strategy for microbial competition in *Streptomyces* spp. [22]. More broadly, analyses of co-localized BGC pairs suggest that physically linked biosynthetic systems may also be associated with nutrient acquisition and oxidative stress protection, although many of these cases remain bioinformatic predictions rather than experimentally validated examples [30]. Among the known functional outcomes of bacterial BS, synergistic antimicrobial activity underscores the potential of these systems as naturally optimized platforms for the development of antimicrobial combined therapies.

**Figure 2:**
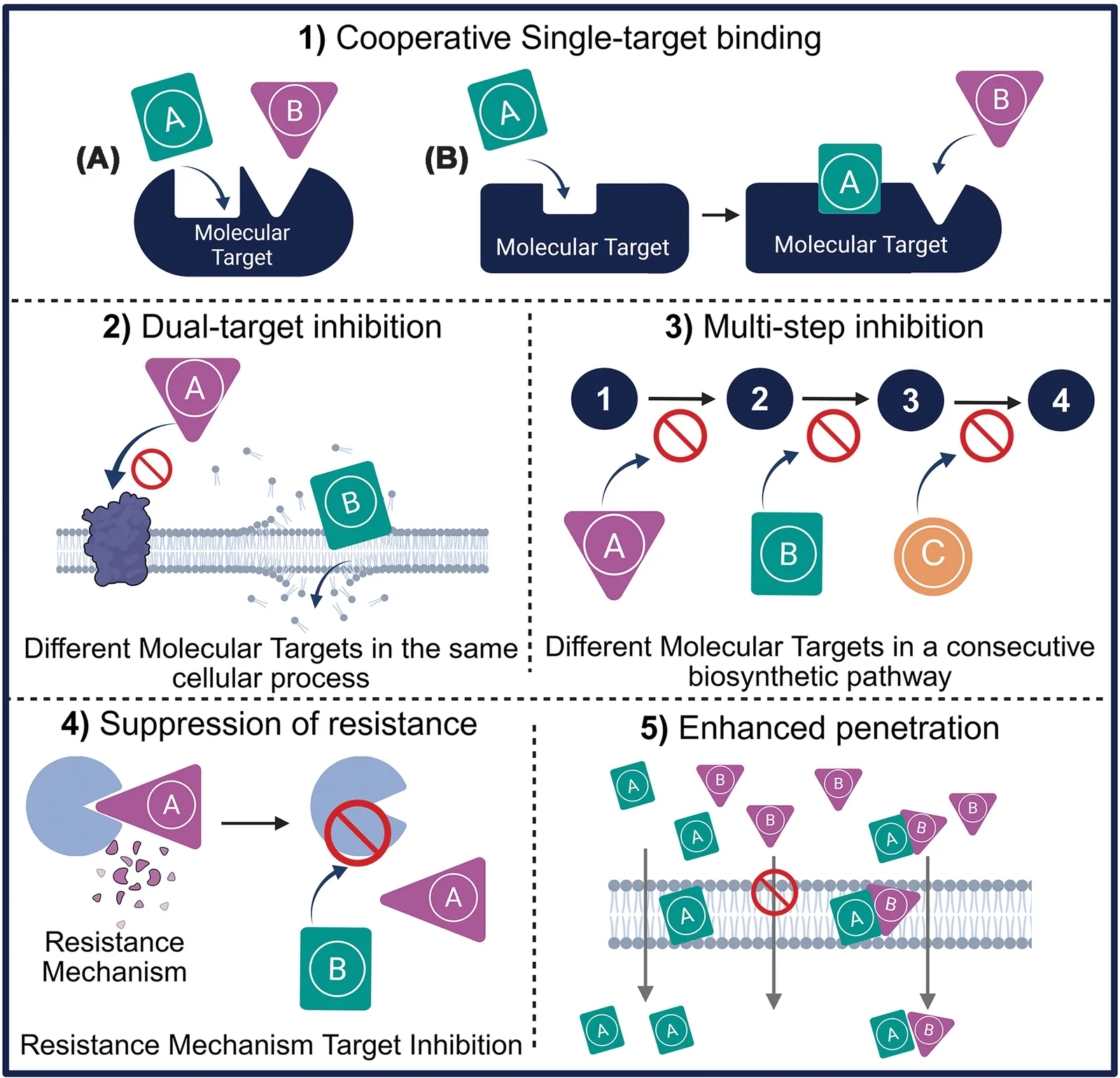
Synergistic mechanisms displayed by supercluster-derived metabolites Schematic overview of the principal mechanism by which metabolites co-produced from BSs may elicit synergistic antimicrobial effects: **1**) cooperative single-target engagement, where (**A**) two metabolites jointly bind and (**B**) enhance each other’s affinity for the same molecular target, **2**) dual-target inhibition within a shared cellular process, **3**) multi-step inhibition across consecutive pathway nodes, **4**) suppression of resistance mechanisms, and **5**) enhanced intracellular penetration of co-produced compounds.

**Figure 3:**
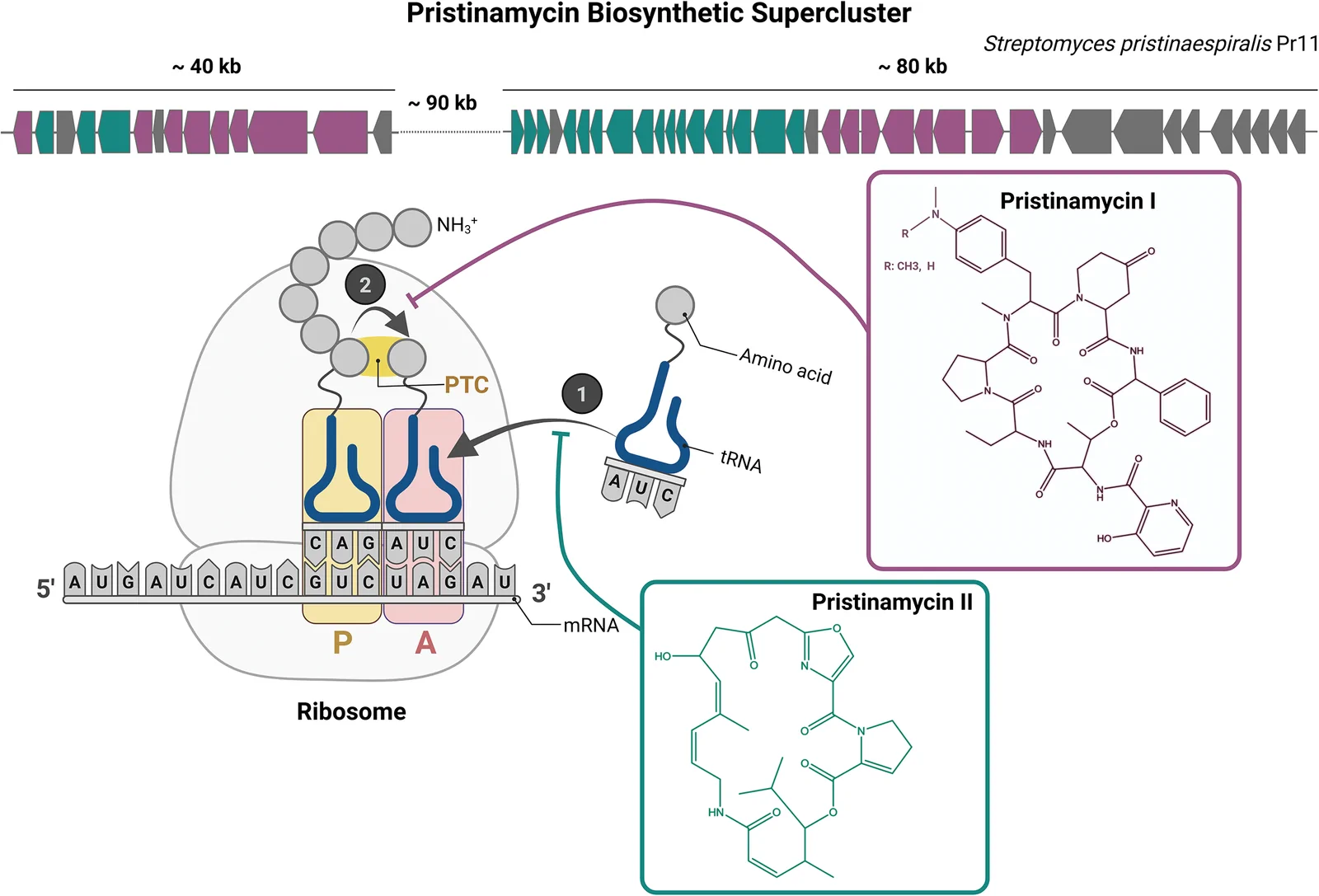
Pristinamycin supercluster and cooperative inhibition of ribosomal function Schematic representation of the pristinamycin supercluster from *Streptomyces pristinaespiralis* Pr11, together with the chemical structures of the co-produced compounds pristinamycin I (PI) and pristinamycin II (PII), and a simplified overview of their cooperative inhibition of ribosomal function. PI and PII biosynthetic genes are shown as purple and green arrows, respectively, whereas genes involved in regulation, resistance, or unknown functions are shown in gray. The ribosome diagram schematically represents the sequential and cooperative action of pristinamycin II and pristinamycin I during translation inhibition, highlighting the inhibition of aminoacyl-tRNA binding to the A site by PII and the inhibition of peptide elongation by PI.

In supercluster-derived metabolite combinations, several mechanisms underlying such synergistic effects have been described (Figure 2), including: 1) cooperative single-target binding, where metabolites act together on the same target to enhance activity, as seen with translational inhibitors such as streptogramins—where type A compounds remodel the 50S ribosomal subunit to enhance type B affinity [6]—and by the lankamycin–lankacidin pair, which inhibits complementary steps of ribosomal function [50]; 2) dual-target inhibition, where each metabolite blocks a distinct target within the same cellular pathway, as described for rapamycin and actinoplanic acids, jointly inhibiting TOR and farnesyl-transferase in TOR-regulated signaling [18], and sulfazecin–bulgecin acting on penicillin-binding proteins and the lytic transglycosylase Slt in peptidoglycan synthesis [51], or even for the dual action on the same cellular structure, reported for the bacillomycin D–fengycin combination, where two lipopeptides cooperatively disrupt the fungal cell envelope [20]; 3) multi-step inhibition, encompassing either sequential membrane attack, as described for laxaphycin A/B [21] or stepwise inhibition of consecutive enzymes in biotin biosynthesis by the four anti-biotin supercluster-derived metabolites [22]; 4) suppression of resistance, notably the β-lactam antibiotic cephamycin and β-lactamase inhibitor clavulanic acid [52]; and 5) enhanced penetration, in which one metabolite facilitates the cellular uptake of its partner, as observed for jessenipeptin–mupirocin combination and anabaenolysins acting with cyclodextrins [24,25]. Among these examples, Figure 3 illustrates the pristinamycin system, including its BS organization and a simplified overview of the cooperative inhibition of ribosomal function by pristinamycin I and II. More broadly, recent genome-wide analyses suggest that some membrane-permeabilizing metabolites encoded by co-localized BGCs may function as antimicrobial potentiators, a concept supported by the validation of the polycyclic tetramate macrolactam ikarugamycin as a membrane-active adjuvant, without yet constituting a valid BS [30].

For some supercluster-derived combinations, the mechanistic basis of synergy is inferred primarily from the known modes of action of the individual metabolites [26–29], whereas in other cases it is supported by biocontrol assays using pathway-specific mutants rather than by quantitative synergy assays with purified compounds [20]. For the *Streptomyces* sp. VB1 supercluster, which encodes for the co-production of arylomycins, globomycin, and siamycin I, the potential synergistic mechanism proposed by the authors has been rationalized by their ability to target distinct processes associated with bacterial envelope biogenesis, suggesting that their co-production may enable coordinated disruption of cell-envelope assembly (Figure 2.2) [28]. Similar speculation can be considered for the borregomycin-anthrabenzoxocinone supercluster case [27], for which evidence from related compound families suggests that indolocarbazole/indolotryptoline metabolites may interfere with ATP-binding proteins [53], whereas anthrabenzoxocinones have been linked to inhibition of type II fatty acid synthesis [54], raising the possibility of functional complementarity between the disruption of ATP-dependent cellular processes and inhibition of membrane lipid biosynthesis. The clostrisin–cellulosin supercluster offers a more speculative case, as both peptides were inferred to interact with the membrane of *S. epidermidis* [29]; thus, their co-production may plausibly enhance membrane perturbation, although their precise targets and any coordinated mechanism remain unresolved. Finally, for the petrichorins A/B supercluster, no molecular targets have yet been identified, and the available evidence appears more consistent with enhanced bioactivity linked to dimeric structure than with a defined target-level synergistic mechanism [26].

Understanding how bacterial BSs have evolved may provide a conceptual bridge between the diversification of metabolite portfolios and the development of new strategies to counteract AMR [55]. Natural selection appears to favor the maintenance of multiple biosynthetic pathways whose encoded combined metabolites act synergistically or contingently to enhance ecological fitness, suggesting that selection often targets ensembles of metabolites rather than individual compounds [11,47,56,57]. Comparative genomic analyses further support the hypothesis that individual BGCs often operate as “gene collectives”—co-evolving modules whose enzymes, transporters, and regulators are gained, diversified, or lost as functional units because their combined metabolic output confers greater ecological benefit than any single component [56,58,59]. By extension, superclusters may represent second-order gene collectives, composed of BGC units whose physical clustering facilitates co-production and coordinated regulation across pathways, while also helping preserve functionally advantageous metabolite combinations by reducing the likelihood that recombination, genomic rearrangements, or independent gene loss events uncouple linked biosynthetic components during genome evolution [47]. In this framework, the evolutionary fate of a BS depends on the ecological value of its combined chemistry [11,13]. In this sense, evidence supports a reciprocal process in which functional synergy favors co-retention and coordinated regulation, while physical linkage provides a genetic framework that promotes cross-pathway interactions, modular innovation, and the evolution of chemically integrated metabolite ensembles [11,13,47,56,57].

Drawing on evolutionary analyses, mechanistic insights from individual BGCs can be extrapolated to BS, which likely form through a combination of tandem duplication and divergence of ancestral pathways, homologous recombination between repetitive motifs, insertion of mobile elements, and horizontal acquisition into pre-existing secondary metabolite gene cluster islands, generating multi-BGC *loci* that are subsequently “wired” into shared regulatory circuits [47,56,57,60]. For example, evolutionary analyses of streptogramin producers indicate that types A and B pathways—now intermingled within a supercluster—originate from two ancestral BGCs with high gene sequence homology and conserved organization [15,61]. In *S. pristinaespiralis*, the presence of transposon-like elements within the pristinamycin supercluster further suggests that probably one pathway was acquired by horizontal gene transfer and later fused with the other, resulting in an integrated supercluster that enables coordinated regulation and production of compounds in synergistic ratios [61]. Likewise, the β-lactam supercluster exhibits signatures of origin through ancestral β-lactam BGC duplication and divergence, subsequent incorporation into a recombination-rich genetic island, and eventual consolidation under shared regulators such as CcaR, illustrating how structural adjacency and regulatory consolidation can stabilize multi-BGC systems over long evolutionary timescales [11]. In contrast, the anthrabenzoxocinone–borregomycin supercluster in *Streptomyces* sp. MST-144321 strain illustrates supercluster formation driven by cooperative biosynthesis, where an ancestrally promiscuous cytochrome P450 enzyme links two biosynthetic routes, effectively nucleating a functionally integrated multi-BGC assembly and promoting chemical diversification [27]. Taken together, these examples support a unified evolutionary framework in which bacterial BSs are not accidental gene aggregations but rather evolutionarily assembled and chemically coordinated gene collectives shaped by ecological selection, horizontal gene transfer, genomic rearrangement, and regulatory integration [11,57].

These naturally evolved synergistic metabolite combinations—whether encoded by BS or reflecting analogous mechanisms—have inspired clinically successful antimicrobial therapies. The most prominent example is the streptogramin class, for which two synergistic combinations are currently approved for human use: oral pristinamycin (Pyostacine^®^), used for decades in France, Tunisia, and Lithuania, and the injectable quinupristin–dalfopristin (Synercid^®^), approved in the United States and Europe for multidrug-resistant Gram-positive infections [6]. Likewise, β-lactam/β-lactamase inhibitor combinations—including the clinically established Augmentin^®^ and Timentin^®^, as well as more recently developed combinations currently under clinical phases—exploit the same resistance-suppression mechanism encoded by the β-lactam supercluster [62]. Although no further supercluster-derived combinations have reached clinical practice, this is an active and growing field of research.

## Mining and engineering superclusters for novel antimicrobial combinations

The growing availability of high-quality bacterial genomes, combined with advances in BGC detection algorithms [10], has expanded the landscape of specialized metabolism far beyond what traditional single-metabolite discovery approaches could capture. Classical NP discovery workflows—particularly bioactivity-guided attempts—were optimized to isolate single compounds, inherently separating co-produced metabolites and masking synergistic or contingent partners. This long-standing bias contributed to decades of dereplication-driven discovery and to a systematic underestimation of combinatorial chemistry encoded in bacterial genomes; consequently, fully exploiting the therapeutic potential of BS requires analytical frameworks that move beyond canonical, single-BGC annotation strategies [11]. Modern genome mining pipelines can overcome these limitations. Tools such as antiSMASH [63], PRISM [64], cblaster [65], and DeepBGC [66] provide high-confidence BGC classification, while BiG-SCAPE and CORASON help to delineate their evolutionary relationships across taxa [67]. Deep long-read assemblies [68] and improved boundary predictions [50] further help to identify multi-BGC regions that were previously fragmented or misannotated in draft genomes. Complementarily, LC–HRMS/MS metabolomics, integrated with Feature-Based Molecular Networking [69] and NPLinker [70], helps resolve clusters of structurally related features, facilitating the detection of co-produced molecular families consistent with coordinated activity of adjacent BGCs.

Integrative genomics–metabolomics approaches have substantially advanced the structural and functional characterization of known BS, revealing their evolutionary context and the coordinated production of their metabolites across strains and taxa. In the β-lactam supercluster, for example, early mapping of adjacent clavulanic acid and cephamycin C pathways [14] has been refined by multi-omics datasets—combining genome sequencing, transcriptomic, and metabolomic profiling—clarifying its organization and conservation among multiple *Streptomyces* species, and expose an interconnected regulatory-metabolic network underpinning co-production of a β-lactam and its β-lactamase inhibitor at clinically relevant ratios [23,71–73]. In other reported cases, such as the rapamycin–actinoplanic acid and jessenipeptin-mupirocin superclusters, genome mining coupled to targeted LC–HRMS/MS and feature-based molecular networking has helped to link neighboring BGCs that encode enzymes responsible for the consistent co-production of metabolite pairs, turning simple genomic adjacency into mechanistically validated BS with documented multi-target activity [18,24]. Metabolo-genomic analyses of the marine strain *Streptomyces* sp. VB1 have likewise identified a supercluster integrating the arylomycin, globomycin, and siamycin BGCs into a single multi-BGC region that co-produces these metabolites under the same fermentation conditions and displays potent inhibitory activity against Gram-positive bacteria, illustrating how these pipelines can uncover previously unrecognized supercluster architectures in ecologically relevant isolates [28]. At the genus-wide level, integrated large-scale genome mining and LC–MS/MS-based metabolomic analyses in *Streptomyces* revealed a supercluster in which four convergent BGCs, and flanking streptavidin biosynthetic genes, encode an arsenal of biotin-targeting metabolites, providing an example of how integrative pipelines can uncover conserved, genomically encoded multi-target systems with direct anti-AMR relevance [22]. Complementing this, a genome-wide survey identified 38 co-localized BGC pairs across multiple bacterial phyla, highlighting physical co-localization as a promising criterion for genome-guided discovery of functionally linked NPs [30]. Importantly, phylogenetic and statistical analyses can further strengthen this approach by helping distinguish genuine functional associations from coincidental co-localization.

Experimental validation and engineering of BS rely on combining targeted genetic engineering with high-resolution metabolomics to dissect and rewire multi-BGC *loci*, thereby positioning them as especially attractive scaffolds for next-generation antimicrobial combinations in which multiple pathways can be tuned from a single genomic region [13,74]. In this context, strategies originally developed to activate or remodel single BGCs, such as genetic engineering approaches—promoter engineering, transcription factor cloning, ribosome engineering, and heterologous expression of target BGCs [74], as well as metabolic and process engineering strategies [75], can be applied to multi-BGC superclusters. In the case of β-lactams, omics-guided engineering and bioprocess optimization of *S. clavuligerus* have been used to fine-tune cephamycin C–clavulanic acid production, with interventions spanning media and feed design, precursor flux redirection, and manipulation of cluster-situated and global regulators—most notably the CcaR SARP activator—yielding multi-fold increases in production titers [72]. Another example is provided by the pristinamycin supercluster, where metabolic engineering—through targeted inactivation of the pristinamycin II pathway with overexpression of positive regulators—redirected flux toward pristinamycin I, increasing its production by ∼2.4-fold. This illustrates how supercluster architecture can be exploited to bias output toward a desired partner in a synergistic pair [76]. Additionally, such strategies are increasingly applied to elucidate pathway logic within superclusters, as demonstrated for the anti-biotin supercluster, where refactoring individual subclusters into engineered plasmids and their heterologous expression confirmed both the structure of the supercluster and the co-produced metabolites [22].

## Concluding remarks

Understanding how mixtures of specialized metabolites act in concert to achieve a given biological effect will be central to tackling the escalating crisis of AMR, reflecting the principle that in nature, selection frequently acts on ensembles of compounds rather than on single molecules. BSs exemplify this principle at the genomic level: they encode portfolios of metabolites that often display synergistic or contingent interactions, are co-regulated in response to specific ecological cues, and have been evolutionarily assembled as chemically coordinated gene collectives rather than as accidental genomic aggregations.

Looking ahead, three major challenges stand out. First, there is a pressing need for clear operational criteria and systematic surveys to distinguish genuinely integrated superclusters from *loci* that merely juxtapose unrelated BGCs. Second, most candidate systems still lack rigorous experimental validation of synergy, contingency, and *in vivo* relevance, calling for standardized combination assays, quantitative pharmacodynamics, and mechanistic studies that directly link genomic architecture to interaction profiles. Third, translating these insights into therapeutics will require integrating supercluster-focused genome mining with multi-omics, structural biology, and modern genetic and metabolic engineering so that naturally evolved combinations can not only be discovered but also reprogrammed, diversified, and optimized.

If these conceptual and technical gaps are addressed, BSs may shift from being viewed as evolutionary curiosities to serving as rational entry points for designing modular, evolvable, multi-target antimicrobial ensembles—providing a blueprint for future antibiotic discovery pipelines to harness the strategies that bacteria have evolved for millions of years to survive in competitive, polymicrobial environments.

## Summary

- Bacterial BSs are an emerging and promising source of naturally synergistic antimicrobial combinations.
- They operate as integrated metabolic units shaped by evolution to co-produce functionally complementary metabolites.
- Integrating genome–metabolome mining with modern engineering tools will be key to unlocking and redesigning these systems for next-generation antimicrobial therapies.

## Open Access

This article has been published open access under our Subscribe to Open programme, made possible through the support of our subscribing institutions, learn more here: https://portlandpress.com/pages/open_access_options_and_prices#conditional.

## Abbreviations

AMR: Antimicrobial Resistance
BGCs: Biosynthetic Gene Clusters
BS: Biosynthetic Superclusters
CSRs: Cluster-situated Regulators
NPs: Natural Products
NRPSs: Non-ribosomal Peptide Synthetases
PKSs: Polyketide Synthases
RiPP: Ribosomally synthesized and Post-translationally modified Peptide
PI: Pristinamycin I
PII: Pristinamycin II
